# The Influence of *p*CO_2_-Driven Ocean Acidification on Open Ocean Bacterial Communities during A Short-Term Microcosm Experiment in the Eastern Tropical South Pacific (ETSP) off Northern Chile

**DOI:** 10.3390/microorganisms8121924

**Published:** 2020-12-04

**Authors:** Paulina Aguayo, Víctor L. Campos, Carlos Henríquez, Francisca Olivares, Rodrigo De Ia Iglesia, Osvaldo Ulloa, Cristian A. Vargas

**Affiliations:** 1 Department of Aquatic System, Faculty of Environmental Sciences & Environmental Sciences Center EULA Chile, Universidad de Concepción, Concepción 4070386, Chile; crvargas@udec.cl; 2 Millennium Institute of Oceanography (IMO), Universidad de Concepción, Concepción 4070386, Chile; frolivares@udec.cl (F.O.); oulloa@udec.cl (O.U.); 3 Institute of Natural Resources, Faculty of Veterinary medicine and agronomy, Universidad de Las Américas, Sede Concepcion, Chacabuco 539, Concepcion 3349001, Chile; 4 Environmental Microbiology Laboratory, Department of Microbiology, Faculty of Biological Sciences, Universidad de Concepción, Concepción 4070386, Chile; vcampos@udec.cl; 5 Laboratorio de Fisología y Genética Marina (FIGEMACentro de Estudios Avanzados en Zonas Áridas (CEAZA), Coquimbo 1780000, Chile; carlos.herriquez@ceaza.cl; 6 Facultad de Ciencias del Mar, Universidad Católica del Norte, Coquimbo 1780000, Chile; 7 Department of Oceanography, Universidad de Concepción, Concepción 4070386, Chile; 8 Department of Molecular Genetics and Microbiology, Pontificia Universidad Católica de Chile, Casilla 160-C, Santiago 8320000, Chile; rdelaiglesia@bio.puc.cl; 9 Center for the Study of Multiple Drivers on Marine Socio-Ecological Systems (MUSELS), Universidad de Concepción, Concepción 4070386, Chile

**Keywords:** ocean acidification, bacterial composition, microcosm, bacterial diversity, metagenomics

## Abstract

Due to the increasing anthropogenic CO_2_ emissions, Ocean Acidification (OA) is progressing rapidly around the world. Despite the major role that microorganisms play on the marine biogeochemical cycling and ecosystem functioning, the response of bacterial communities upon OA scenarios is still not well understood. Here, we have conducted a detailed characterization of the composition and relative abundance of bacterial communities in the water column of an open-ocean station in the Eastern Tropical South Pacific (ETSP) off northern Chile and their interactions with environmental factors. In addition, through a short-term microcosm experiment, we have assessed the effect of low pH/high *p*CO_2_ conditions over the abundance and genetic diversity of bacterial communities. Our results evidence a clear partitioning of community composition that could be attributed mostly to dissolved oxygen. However, our experimental approach demonstrated that low pH/high *p*CO_2_ conditions might modify the structure of the bacterial community, evidencing that small changes in pH may impact significantly the abundance and diversity of key microorganisms. This study constitutes a first step aiming to provide insight about the influence of changing carbonate chemistry conditions on natural bacterial communities and to shed light on the potential impact of OA in biogeochemical cycles on the ETSP region.

## 1. Introduction

Little is known on how bacterial communities in the surface open ocean might respond to future changes in *p*CO_2_ and pH conditions [1]. Although the ocean has partially absorbed a significant fraction of the anthropogenic atmospheric CO_2_ emissions, this has come at the expense of altering carbon chemistry on the global ocean, disturbing its delicate geochemical balance [2]. This disruption of carbonate chemistry processes is known collectively as Ocean Acidification (OA) [3]. This disruption by the net effect of CO_2_ dissolution in the ocean causes an increase of hydrogen and bicarbonate ion concentration, altering seawater pH, and decreasing carbonate ions [4], which has profound consequences for marine biodiversity and ecosystem functioning in coastal and open ocean waters [5,6].

A variety of laboratory experiments have been conducted aiming to evaluate the impact of OA in different marine organisms, including photosynthesis, net growth, and ingestion mainly in organisms and microorganisms with calcareous structures, calcification, reproduction, behavior, gene expression, and biological interactions, among others [7,8]. Nevertheless, the proportion of experimental studies conducted with naturally occurring communities in open ocean waters is relatively scarce. Since OA might impair some highly relevant functional groups of marine ecosystems, such as planktonic organisms [9] and small microorganisms [10], it may have potential implications in highly relevant biogeochemical processes in the open ocean, such as altering the nitrogen fixation, denitrification, and regeneration of organic carbon [11] and nitrification [12]. Beman et al. [12] showed several lines of evidence indicating that nitrifying microorganisms may be sensitive to future changes in seawater pH or *p*CO_2_ levels. However, cultivated strains of ammonia-oxidizing bacteria (AOB) contain putative carbon concentrating mechanisms (CCMs) that could reduce their sensitivity to changes in *p*CO_2_ [10]. For instance, it is well known that *Bacteria* and *Archaea* mostly drive nutrient cycling in the ocean [7]. Recent studies suggest that contemporary evolution could support maintaining the functionality of microbial processes in the ocean in the face of global change [13,14].

Due to logistical reasons, manipulation experiments of changing pH/*p*CO_2_ conditions are difficult to implement during research cruises. Even so, some experiments have been carried out, and the response of bacterioplankton in terms of community composition and activity to reductions in seawater pH varies considerably between experimental studies linked to the different waters masses and seasons [14,15]. Changing microbial community structure caused e.g., by microbial responses to OA and warming may also have significant implications in the balance between autotrophic production and heterotrophic degradation processes in the surface ocean, which can result in a powerful feedback effect on atmospheric CO_2_ concentrations [15,16]. The risks of OA may vary greatly among marine ecosystems. While open ocean ecosystems are characterized by a certain homogeneity in terms of pH levels (8.0–8.1) [17], coastal ecosystems are characterized by a large degree of pH variability (7.4–8.1), which is mostly driven by mesoscale processes such as the effect of freshwater discharges [18] and coastal upwelling injection of corrosive waters with low pH and highly supersaturated in CO_2_ [19]. These conditions determine that coastal vs. ocean communities have a contrasting environmental history of exposure to different pH/*p*CO_2_ levels. These differences allow us to hypothesize that marine bacterial communities may have different levels of sensitivity or tolerance to changes in pH/*p*CO_2_ based on their physiological resilience, which is determined by its environmental exposition to homogeneous or heterogeneous pH/*p*CO_2_ conditions.

In the present study, we provide insights into the characterization of the composition and relative abundance of water column bacterial communities in a sampling site at the ETSP off northern Chile, as well as the associated environmental conditions, including nutrients (NO_2_^−^_,_ NO_3−_, PO_4_^3−^), and especially carbonate chemistry conditions (total alkalinity, AT; pH_T_; dissolved inorganic carbon, DIC; and carbon dioxide partial pressure, *p*CO_2_). Furthermore, we evaluated the response of surface bacterial communities to potential OA scenarios by using on-board short-term microcosm *p*CO_2_–perturbation experiments. We hypothesize that the microbial community structure in Open Ocean ecosystems can be modified upon high *p*CO_2_ conditions. For this purpose, we have conducted a 16S rRNA gene sequencing analysis focused on evaluating the taxonomic response of oceanic marine bacterial communities to experimental low pH/high *p*CO_2_ conditions. Our results evidence that small changes on pH may impact significantly the abundance of key microorganisms participating in the nitrogen cycle upon low oxygen conditions. The present study constitutes a first step aiming to provide insight about the consequences of OA on natural bacterial communities and therefore, to shed light on the potential impact of OA in biogeochemical cycles on the ETSP region.

## 2. Materials and Methods

### 2.1. Study Area

**Figure 1:**
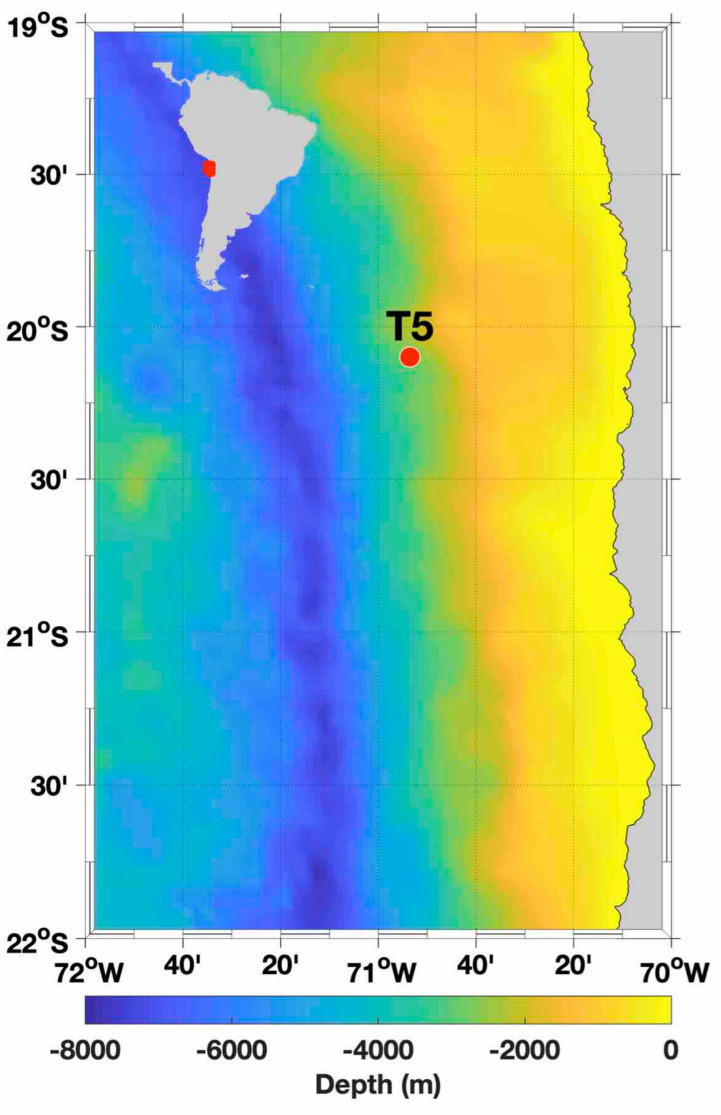
Study site on the continental shelf in northern Chile. The yellow circuits indicate the stations considered in this study: T5 stations in the Lowphox I cruise.

Sampling and experiments were carried out during an expedition to the ETSP off northern Chile between 20 November and 14 December 2015 on-board the R/V Cabo de Hornos (Figure 1). Seawater was sampled at a site located around 43,496 nautical miles (Station 5; 20°05′ S, 70°53′ W) offshore. The sampling station was selected based on its biogeochemical characteristics, such as the presence of occasional anoxia in subsurface waters and the accumulation of NO_2_^−^ upon anoxic conditions (AMZ) [20]. At this site, the Peru-Chile undercurrent transports O2-deficient equatorial subsurface water southward, and the O_2_-rich surface water is transported north by the Humboldt Current [20].

### 2.2. Seawater Collection

Seawater samples for nutrients, pH_T_, and total alkalinity (AT) were collected at −1, −25, −50, −100, −200, −300, and −400 m depth by using a Seabird SBE-911 CTD system (CTD: Conductivity, Temperature and Depth), which was equipped with a SBE 43 oxygen sensor and fluorometer, and an oceanographic rosette equipped equipped with 10 L Niskin bottles (General Oceanic^®^, Miami, Florida, FL, USA).

For DNA analyses of T5 station, 5 L of seawater were taken at selected depths (−20, −50, −73, −160, −250, and −300 m depth). Seawater was pre-filtered through a 20-mm pore size mesh and through a 3-µm pore size GSWP membrane (particle-attached fraction (PA), and the microbial biomass was collected onto a 47-mm-diameter GTTP membrane with a 0.2-µm-pore size (Free living size fraction (FL)). Then, both filters were transferred to cryovials and covered with DNA lysis buffer (40 mmol L^−1^ ethylenediaminetetraacetic acid (EDTA), 0.73 mol L^−1^ sucrose, and 50 mmol L^−1^ Tris-HCl, pH 8.3), immediately frozen in liquid nitrogen, and stored at −20 °C until DNA extraction.

### 2.3. Physical-Chemical Analyses

Temperature (T), salinity (S), and oxygen concentrations were measured through the upper 350 m of the water column using a high-resolution customized Pumping Profiling System (PPS) aligned in real-time with a Sea-Bird 25 CTD equipped with a SBE43 oxygen sensor (Bellevue, WA, USA). The PPS pumped at a rate of 2.7 L min^−1^ and had a pump-to-deck time of 270 s.

pH_T_ samples were collected with 50 mL syringes and immediately transferred to a 25 mL thermostated cell at 25.0 ± 0.1 °C for standardization, with a pH meter (Metrohm^®^, model 6.0258.600, Herisau, Suiza) using a glass combined double junction Ag/AgCl electrode) calibrated with 8.089 Tris buffer solution as a certified reference material (CRM, supplied by Andrew Dickson, Scripps Institution of Oceanography, San Diego, CA, USA) at 25 °C. pH values are reported on total scale (pH_T_). Samples for A_T_ were poisoned with 50 µL of saturated HgCl_2_ solution and stored in 500 mL borosilicate BOD bottles with ground-glass stoppers lightly coated with Apiezon L^®^ grease and kept in darkness and room temperature. A_T_ was determined using the open-cell titration method [21] by using an automatic Alkalinity Tritrator (AS-ALK2 Apollo SciTech^®^, Newark, NJ, USA). All samples were analyzed at 25 °C (±0.1 °C) with temperature regulation using a water bath. The accuracy was controlled against a certified reference material (CRM, supplied by Andrew Dickson, Scripps Institution of Oceanography, San Diego, CA, USA) and the A_T_ repeatability averaged 2–3 µmol kg^−1^. Temperature and salinity data were used to calculate *p*CO_2_ by using CO2SYS software for MS Excel [22] set with Mehrbach solubility constants [23] refitted by Dickson and Millero [24]. The KHSO_4_ equilibrium constant determined by Dickson [25] was used for all calculations.

The determination of nitrite (NO_2_^−^), nitrate (NO_3_^−^), and phosphate (PO_4_^3−^) concentrations were conducted from seawater samples collected in 15 mL polyethylene flasks and stored at –20 °C until further analysis. Analyses were conducted upon World Ocean Circulation Experiment (WOCE) protocol by using a standard colorimetric technique in a segmented flow Seal AutoAnalyzer (Seal Analytical AA3, Wisconsin, WI, USA) [20]. Analytical detection limit to NO_3_^−^ + NO_2_^−^ and PO_4_^3−^ were 0.05 μM and 0.02 μM, respectively.

### 2.4. Cell Abundance Estimates

Seawater samples for flow cytometry were taken each 5.8 m and analyzed on-board using a “jet-in-air” Influx Mariner Flow cytometer to detect and enumerate microorganisms (total eukaryotic phytoplankton, heterotrophic Bacteria, and Archaea). Total eukaryotic phytoplankton was identified by their red fluorescence (692/40 nm) as a proxy of chlorophyll-a by using a combination of 457, 532, and 640 nm excitation lasers. *Synechococcus* was discriminated against eukaryotic cells by forward angle light scatter (FALS) and the orange phycoerythrin fluorescence (580/20 nm) using a 457 nm and 532 nm excitation lasers. For auto-fluorescence detection, events were triggered on the FALS. For the detection and enumeration of heterotrophs, cells were stained with Sybr Green as described in Marie [26] and detected by the green fluorescence (530/15 nm) and FALS using a 488 nm laser. For the autotrophic fraction, 100 μL of each sample was run at a constant flow rate of 20 μL/min. For heterotrophs, 70 μL of each sample was run at a flow rate of 30 μL min^−1^. Flow rate was monitored by using a flowmeter (Sensirion, Staefa ZH, Switzerland). The Spigot software (Model v6.x, Bangalore, India) was used for event recording and the FlowJo software (FlowJo LLC v7.6.1, Oregon, OR, USA) was used for data analysis.

### 2.5. Short-Term pCO2 Perturbation Experiments

#### 2.5.1. Microcosms Experiments

First, 40 L of surface seawater collected through a CTD-rosette sampler from 10 m depths (Station T5) was gently pre-filtered on-board through a 200 μm cut-off nylon net to exclude big particles and zooplankton. Subsequently, larger microorganisms were removed by filtration through 10 μm IsoporeTM Membrane Filters (TCTP-type, Millipore, Eschborn, Germany). The seawater was stored in the dark in sterile (autoclaved) 4 L polycarbonate bottles until the experiment setup (less than 2 h).

Experimental setup considered three replicate bottles for each pH*/p*CO_2_ treatment ≈400 and 1200 μatm (for pH_T_ and pCO_2_ respectively) and two control bottles, without any kind of CO_2_ manipulation (10 bottles in total). The duplicate control bottles aimed to evaluate only the “bottle effect” in order to analyze the changes in microbial communities caused by other effects than air and/or CO_2_ manipulation.

Similarly, initial samples also were collected from each carboy. This was regulated by the manipulation of environmental CO_2_ levels. pH was maintained in each carboy using a computerized control system (AquaMedic, Bissendorf, Germany) attached to solenoid valves and CO_2_-gas bottles. The pH levels were regulated by addition of pure gaseous CO_2_ directly into the experimental bottles to a resolution of 0.04 ± 0.005 pH units, and the pH-system controller and solenoid valves controlled the bubbling. To avoid hypoxic and anoxic conditions due to the release of pure CO_2_, all experimental bottles were bubbled with CO_2_-free atmospheric air.

Incubation was conducted on deck in a temperature-controlled water-circulating tank for 5 days under an in situ surface temperature of 20 to 21 °C. Subsamples were collected from each incubation bottle every day during the course of the experiment. Samples for pH measurements were collected in 50 mL syringes, and pH values for each treatment were determined as previously mentioned.

#### 2.5.2. Metagenomic Analyses

For DNA extraction, 2 L of seawater from each microcosms were collected at the end day of the experiment, pre-filtered through a 20 µm pore size mesh, and passed sequentially through a 3 µm, and the biomass was collected onto 0.22 µm pore size PES filters as described for the natural communities. DNA was extracted as previously described by Murillo et al. [27]. T5 station was used as control, according to procedure describe above.

Sequences were obtained by amplification and massive sequencing of the 16S rRNA gene at the Roy J. Carver Biotechnology Center at the University of Illinois, using the fluidigm technology for amplification and Illumina 2 × 250 nt Miseq2000 as the sequencing platform. The primer pair 515F and 806R were used for the amplification of the hyper-variable V4 region of the Bacterial 16S rRNA gene. The sequences obtained were analyzed using the USEARCH sequence analysis tool (v10.x). Briefly, sequences were paired, trimmed, and filtered. Operational taxonomic units (OTUs) were defined according to 97% of sequence similarity. Clean sequences were aligned locally against the SILVA database (v.128, Bremen, Germany) [28], using the BLAST tool (MEGABLAST, values < 0.001 and bitscore > 50) [29]. The taxonomy was assigned to the order level according to SILVA classification and manual curation based on other work done in similar study areas [20,30]. Sequences that did not correspond to bacteria and single sequences (singletons) were removed. All the sequences were submitted to GenBank with the following numbers SAMN10794715 to SAMN10794734. The community structure was determined based on the composition and relative abundance of the different taxa present in each sample. The R environment was used to graphically represent the relative abundances, considering only the abundant taxa (relative abundance >1% of the community in all the samples). For diversity analysis, samples were rarefied to the lowest number of reads in the whole dataset and the different diversity indexes (Shannon diversity (H’), Evenness index were obtained using USEARCH. To compare the bacterial community compositions across groups of samples, Bray–Curtis similarity analyses were performed, and similarity matrices were used to obtain a CLUSTER graph, by using PRIMER 6.1.18 (Primer-E, Ltd. Albany, New Zealand).

### 2.6. Statistical Analysis

The experimental data were recorded using three replicates (*n* = 3) and data were expressed as mean ± SD. Data were analyzed using two-way ANOVA and Tukey’s multiple comparison test. *p* values < 0.05 were considered as statistically significant. Bio-Env Analysis (Primer-E, Plymouth, UK) and Redundancy Analysis (RDA) (XLSTAT-ADA, Addinsoft SARL, Paris, France) were also used to determine the level of correlation of the bacterial community composition with environmental variables such as dissolved oxygen, temperature, TA, salinity, pH, *p*CO_2_, nutrients, and DIC, in order to assess the extent of the contribution of each variable to the variability in the community composition.

## 3. Results

### 3.1. Physical–Chemical Conditions and Non-Pigmented Picoplanktonic Cells

**Figure 2:**
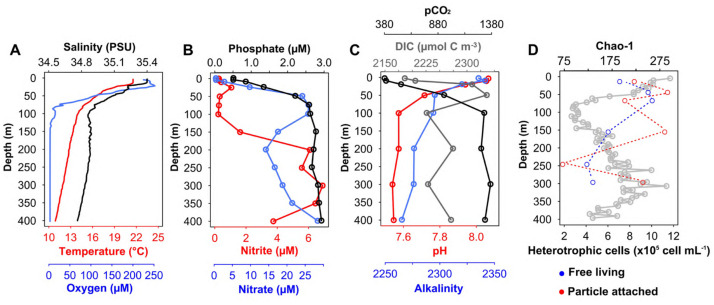
Characterization of the environmental variables: (**A**) Dissolved oxygen, temperature, salinity; (**B**) Nitrate, nitrite, and phosphate (**C**) pH, *p*CO_2_ and alkalinity; (**D**) Cellular abundance of the water column of the T5 station.

Vertical profiles of temperature, salinity, dissolved oxygen, nutrients, carbonate chemistry parameters, and non-pigmented picoplanktonic cells from station T5 are shown in Figure 2. The water column at station T5 was characterized by a clear thermocline and halocline between 10 and 80 m. The concentration of O_2_ was higher at the surface (>240 μM) but falls abruptly from the surface to approximately 70 m (Figure 2A). The sampling station (T5) clearly evidenced an anoxic layer (AMZ) evidenced by the accumulation of NO_2_^−^ (>0.5 μM). The main AMZ core (NO_2_^−^ > 6 μM) was observed between 200 and 350 m depth (Figure 2B). The analysis of the carbonate system in the water column showed the highest pH_T_ values in the surface layer (pH_T_ 8.05), which decrease from surface to 100 m depth, where pH_T_ was relatively constant between 7.4–7.5 until 400 m depth. The vertical distribution of pCO_2_ showed an opposite pattern to that observed for pH_T_, whereas the total alkalinity (A_T_) showed a slow decrease below 50 m to the deepest layer sampled during this study. The highest DIC concentration was observed in the subsurface layer at 50 m depth (≈2325 μM kg^−1^) and minimum (≈2225 μM kg^−1^) at 100 m and at the core of the AMZ zone at 300 m depth (Figure 2C).

Total bacterial cell counts to free living were 10^6^ cells mL^−1^ in each surface layer. At 700 m depth, the cells count decreased to 10^5^ cells mL^−1^. Between 200 and 350 m depth, we can observe a increase in the abundance of cells that reached values in the order of 10^6^ cells mL^−1^ (Figure 2D). There are communities associated with particles, which in high abundance are immersed in these conditions of low pH/low O_2_.

### 3.2. Vertical Distribution and Community Structure of Bacterial Assemblages in Station T5

**Figure 3:**
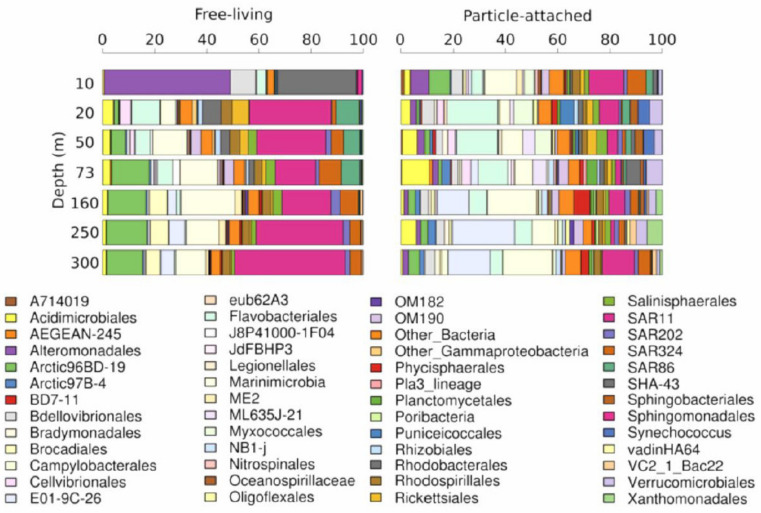
Relative abundance of the most abundant taxa (above 1%) in at least one sampling point at T5 station for two fraction sizes (free-living and particle attached).

The obtained OTUs comprise a total of 166 taxa present through the O_2_ gradient in the water column. From those, 45 taxa were represented in high proportions and considered abundant in at least one depth (Figure 3). In the particle-attached fraction of a surface mixed layer (10 m depth), *Alteromonadales* (9%), Arctic96BD-19 (10%), SAR11 (17%), and SAR324 (9%) were phylogenetic groups with the higher relative abundance of total bacterial sequences. For free-living bacteria samples, *Alteromonadales* (50%), *Bdellovibrionales* (10%), and *Rhodobacterales* (32%) were phylogenetic groups with major abundance with abundances relatives. The clade SAR11 predominates (30–32%) from 20 to 300 m deep in free-living fraction. The Arctic96BD-19, J8P41000-1F04, and SAR324 groups (≈10%) were also highly represented. Under anoxic conditions, *Marinimicrobia* reached maximum relative abundance, together with SAR11 (≈20%), followed by Arctic96BD-19 (16%) and to a lesser extent SAR324 (7.5%) and SAR202 (3.4%). At the core of the AMZ (250 and 300 m), SAR11 was the dominant component of the bacterial community, with a relative abundances of 35 and 44.5%, respectively. *Marinimicrobia* decreased its relative abundance (≈12%), as well as SAR324 (4.5%) and *Acidimicrobiales* (1.4%). For particle-attached bacteria, the most abundant group was the order *Flavobacteriales* (26.4%), followed by SAR11 (11.5%). Moreover, under anoxic conditions, *Marinimicrobia* and E01-9C-26 predominate, instead of *Flavobacteriales*, which decrease their relative abundance. SAR11 is abundant in the upper layer and in the anoxic core, as is SAR324.

BEST analysis (BIO-ENV algorithm, Clarke, 1993) was used to assess which measured environmental variables could explain the observed patterns in the bacterial community structure. Oxygen saturation was the single variable that best explained the bacterial community pattern (Spearman correlation coefficient Rho of 0.647, *p* value = 0.01), while temperature, nutrients, pH, *p*CO_2_, TA, and DIC were the abiotic variables that explained the highest proportion of variance when taken together (Rho = 0.683, *p* value = 0.01) (Table S1). For free-living fraction, phosphorus was single variable that best explained the bacterial community pattern (Spearman correlation coefficient Rho of 0.818, *p* value = 0.01) (Table S2).

### 3.3. Response of Bacterial Communities to Short-Term Exposure to Elevated pCO_2_ Levels in Microcosms Experiment

**Figure 4:**
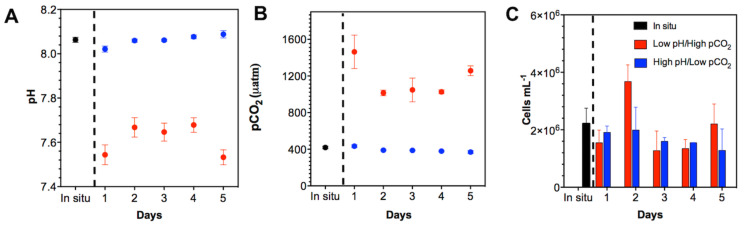
Characterization of the environmental variables in microcosm: (**A**) pH; (**B**) *p*CO_2_; (**C**) Cellular abundance. The black circle represents oceanic water from station T5 (In situ); the blue circle represents the control treatment high pH/low *p*CO_2_, and the red circle represents treatment under pH/high *p*CO_2_.

Seawater carbonate system parameters during field collection (i.e., in situ) and for the experimental setup are given in Figure 4A,B. It is noteworthy that the pH/*p*CO_2_ levels established for the two experimental treatments (low/high) were maintained constant over the 5-day experiments, as reflected by the continuous monitoring of pH and *p*CO_2_ value (Figure 4A,B).

During the experimental period, the carbonate system parameters under simulated CO_2_-driven OA showed significant differences in the pH values and *p*CO_2_concentration between both treatments and control (Figure S1). As expected, no significant differences were found in the total A_T_ and nutrients after five days of incubation (ANOVA *p* > 0.05). Post hoc (Tukey’s test) analysis revealed that there were no significant differences (*p* = 0.019).

In order to compare pH/*p*CO_2_ effects over/on bacterial communities, we analyzed whole data by comparing both pH/*p*CO_2_ treatments with the control microcosms without any kind of carbonate chemistry manipulation and nutrients (Figure S1). Hereafter, we refer to changes in bacterial abundances over time as net bacterial count, which was mostly due to bacterial abundances that could also be regulated by microbial grazing and viral lysis.

After five days of incubation, the net bacterial count was faster under high pH conditions compared to the control conditions (Figure S2). All the microcosms showed an increase in the number of cells on Day 2 (Figure 4C). The highest bacterial abundances were accounted in the high pH/low *p*CO_2_ microcosms; the low pH/high *p*CO_2_ microcosm showed a peaking on Day 2, with values up to 4 × 10^6^ cells mL^−1^. From Day 3, a reduction in bacterial count was observed in all the treatments, which is especially significant in the low pH/high *p*CO_2_ treatment (ANOVA *p* < 0.05). Tukey’s test showed significant differences between low pH and high *p*CO_2_ treatments (*p* = 0.022). Nevertheless, toward the end of this short-term experiment, bacterial abundance reaches 2 × 10^6^ cell mL^−1^ in both high and low pH treatments. After 5 days of incubation, the microcosms with low pH/high *p*CO_2_ and high pH/low *p*CO_2_ showed significant differences in their total bacterial counts as compared with those observed in control microcosm (ANOVA *p* < 0.05). Tukey’s test showed significant differences (*p* = 0.01).

### 3.4. Community Structure of Free-Living and Particle-Attached Bacteria in the pCO_2_ Perturbation Experiments

**Figure 5:**
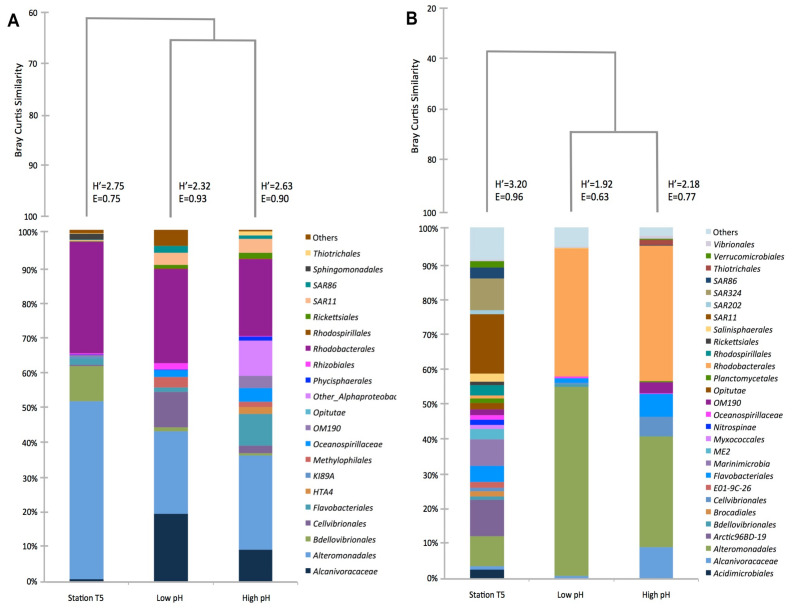
Cluster diagram of relative abundance of the most abundant taxa (above 1% in at least one sampling point in microcosm) assigned to bacterial phylogenetic groups obtained from station T5, treatment high pH/low *p*CO2 and treatment under pH/high *p*CO2 in two fraction sizes: (**A**) Free- living and (**B**) Particle attached), *n* = 3. *H’*: Shannon diversity index, *E*: Evenness index.

The phylogenetic analysis of the V4 16S rRNA sequences showed that the structure of the bacterioplankton community composed of free-living and particle-attached fraction of the microcosm differed, after 5 days of incubation, from that present in the original water sample (T5 station/in situ). Sequences of the dominant taxonomic groups (abundances ≥1%) across all free-living and particle-attached fractions in the microcosm experiments were affiliated to *Alteromonadales* and *Rhodobacterales* (Figure 5A,B). Phylogenetic analysis showed that not all bacterial groups detected in T5 station/in situ were present in microcosms. These results demonstrate that changes in pH and *p*CO_2_ modify the bacterial composition of the ocean, with respect to phylogenetic groups present at the T5 station but absent at the end of the microcosms assays. Only one free living taxon, *Sphingomonadales* (2%), was in this condition, but in the case of particle-adhered bacteria, this was observed for seventeen phyla. These phyla were *Acidimicrobiales* (3%), Arctic96BD-19 (10%), *Brocadiales* (1%), E01-9C-26 (2%), *Marinimicrobia* (8%), ME2 (3%), *Myxococcales* (1%), *Nitrospinae* (1%), *Opitutae* (2%), *Planctomycetales* (1%), *Rhodospirillales* (3%), *Rickettsiales* (1%), *Salinisphaerales* (2%), SAR202 (1%), SAR324 (9%), SAR86 (3%), and *Verrucomicrobiales* (2%).

For free living, in the high pH/low *p*CO2 microcosms, *Alteromonadales* (26%), Other *Alphaproteobacteria* (10%), *Rhodobacterales* (22%,) *Alcanivoracaceae* (9%), and *Flavobacteriales* (9%) were the most abundant phylogenetic groups. In the low pH/high *p*CO2 microcosm, *Rhodobacterales* (27%), *Alteromonadales* (23%), *Alcanivoracaceae* (19%), and *Cellvibrionales* (10%) were the most abundant phylogenetic groups. Regarding particle-attached fractions, the phylogenetic groups showing the higher abundances in the high pH/low *p*CO_2_ microcosms were *Alteromonadales* (31%), *Cellvibrionales* (5%), *Rhodobacterales* (39%), *Alcanivoracaceae* (9%), and *Flavobacteriales* (6%). These results also show that changes in pH/*p*CO_2_ modify the diversity and abundance of bacterial groups present at the beginning of the experiment.

In the low pH/high *p*CO_2_ microcosms, *Rhodobacterales* (36%), *Alteromonadales* (54%), and *Flavobacteriales* (2%) were the most abundant phylogenetic groups. In addition, in both size fractions, the low pH/high *p*CO_2_ microcosm showed a low diversity index. For both size fractions, the most abundant phylogenetic groups retrieved from the control were similar to those present in high pH/low *p*CO_2_ microcosm except for HTA4 *Gamma*–*Proteobacteria*, which was absent in the control (Figure S3).

In addition, a cluster diagram representing similarities in the community structure of free-living and particle-attached fractions of the *p*CO_2_ perturbation experiments was done. This diagram showed that for both the free-living bacterial community and particle-attached bacterial community, T5 station, and high pH/low *p*CO_2_ treatment show a high degree of similarity: over 60%. However, the bacterial community associated with the low pH/high *p*CO_2_ treatment decreased the similarity value to 40%.

## 4. Discussion

Climate changes could produce alterations within marine microorganisms communities composition, ocean acidification, and an expansion of oceanic oxygen minimum zones (OMZs), which have a central role in biogeochemical cycles. In our study, the analysis of bacterial diversity at T5 ocenic station (OMZ) showed that *Marinimicrobia*, E01-9C-26 and SAR324 were phylogenetic groups that predominated in the anoxic zone, and SAR11 was abundant in the upper layer. *Protobacterial* and *marinimicrobial* microbial communities have been reported from other O2-deficient marine environments [31,32]. The members of these groups were found to have the genetic potential for coupling the C, N, and S cycles in the AMZs [31]. In particular, some authors suggest that the role of the bacteria of the SAR324 group in the nitrogen cycle could be related to nitrate as an electron acceptor for the bacteria of the group due to the low concentration of oxygen in the area [33,34].

In particular, understanding the response of marine bacteria to OA is important for evaluating the effects of climate change on the marine ecosystem. OA also affects the microorganisms responsible for the net productivity of the ocean and of the carbon cycle, influencing the cycling and export of organic matter to the deep sea [35]. So far, few studies describe the response of microorganisms to OA. Most of the marine microorganisms are yet unculturable [36]. Therefore, this might be one of the reasons for the reduced exploitation of marine microbes as a biological model for OA studies or other global change-related drivers.

Our work is the first pH/*p*CO_2_ perturbation study in the ETSP region, and moreover, it also was the first full-scale experimental work in this region using this sea-going microcosm system on-board a research vessel. Our results showed an increase in the net bacterial growth due to OA. To some extent, grazing and viral lysis could affect bacterial abundances during our study; nevertheless, beneficial effects of elevated *p*CO_2_ were more evident. Using mesocosms, Endres et al. [20,35] also showed an increase in the net bacterial growth at low pH/high *p*CO_2_ levels, suggesting that the reasons for the enhanced bacterial success were increasing the accumulation of gel particles as the substrate to attach to and a major nutrient availability due to higher rates of cell-specific protein hydrolysis. Grossart et al. [37] also reported increasing bacterial abundances of particle-attached bacteria at low pH/high *p*CO_2_ conditions mostly during the decline of a phytoplankton bloom and the subsequent high release of algal-derived dissolved organic matter. In addition, a study reported by Crespo et al. [38] described that particle-attached bacteria are present in higher concentrations than those found free in the seawater and that they incorporate more substrates and have higher hydrolysis rates than the free-living bacteria.

Previous studies have shown minor effects of *p*CO2-driven OA on bacterial abundance [37]. For instance, Rochelle-Newall et al. [39], using a small mesocosm system, found no pCO_2_-dependent differences in total bacterial abundances. In addition, they were unable to distinguish between free and attached bacteria or between different growth phases, which may account for the observed differences. Decreasing pH in the ocean is a very slow rate process and conducting experiments in simulated conditions at an oceanographic research cruise is also a hard task. The gradual acidification process in nature or under laboratory-controlled conditions will act differently on the physiology of an aquatic ecosystem. Maas et al. [40] studying the response of a bacterial community at the Ross Sea (Antarctica) toward OA concluded that bacterial diversity varies with the incubation period under acidified conditions. Thus, it is speculated that prolonged exposure to pH shifts will substantially modify the microbial composition of the oceans. Our results showed that the taxonomic classification of sequences of major groups (*Alteromonadales*, *Alcanivoracaceae*, *Rhodobacterales*) did not significantly change due to low pH/high *p*CO_2_ conditions. However, cluster similarity analyses (Bray-Curtis) of relative abundances of sequences showed that low pH/high *p*CO_2_ microcosm produced effects on the structure of the bacterial communities, detecting a low percentage similarity when compared to the other microcosms both in particle-attached and free-living bacteria.

The increment in *Alteromonadales* has been previously reported in mesocosms studies. A comparison of the bacterial community in our microcosms using amplification and massive sequencing of the 16S rRNA gene revealed differences in the structure of the bacterial community when manipulating the pH/*p*CO_2_ over time. Therefore, the changes in genetic diversity observed under low pH/high pCO_2_ conditions could be considered as a consequence of the negative impact of the pH/*p*CO_2_ levels change upon specific susceptible bacterial groups and thus modify the bacterial diversity commonly found in the marine ecosystem. Das and Mangwani [14] suggested that higher *p*CO2 and lower pH would cause changes in microbial diversity and composition. In this study, using microcosm assays, we evaluated how changes in pH and *p*CO_2_ modify the bacterial composition of the ocean. Interestingly, in the low pH/high *p*CO_2_ microcosm, SAR 11 showed low relative abundances at the end of the experiment. These organisms are recognized as key players in the nitrogen cycle of AMZs regions [41]. Most studies have shown that in fact, pH changes can also affect biogeochemical cycling processes rather than only microbial diversity [29,42,43].

In addition, Krause et al. [44], using a culture-dependent microcosm approach, reported that even a small pH shift in North Sea waters may have a significant and direct effect on the bacterial community composition and they identified *Gammaproteobacteria*, *Flavobacteriaceae*, *Rhodobacteraceae*, and *Campylobacteraceae* as phylogenetic groups responding with changes in their relative abundances to changing pH conditions. Meron et al. [45] reported that reduced pH levels could also result in an increase in bacterial growth of many pathogenic microorganisms associated with corals, such as *Vibrionaceae* and *Alteromonadaceae*.

The present study contributes to at least understanding the potential impact of OA, a global change driver, which can affect bacterial communities in a so far unexplored region of the ocean. In order to improve our knowledge on the effect of changes in oceanic pH/*p*CO_2_ on the dynamics of bacterial communities, it is necessary to broaden the focus of experiments, including the combined effects of all changes affecting the environment (e.g., temperature, oxygen). Bacterial communities can adapt to the pH/*p*CO_2_ changes but only up to a certain degree before their rate of adaptation plateaus will no longer be able to keep up with the consistent decrease in pH in the next one hundred years. Our results, although preliminary, suggest that small changes in pH/*p*CO_2_ have the potential to cause changes in the bacterial composition of the communities. It can be expected that in long-term perturbation experiments, other taxa of specific bacteria could respond to low pH/high pCO_2_ conditions.

In this work, we compared in situ samples subjected to a pH/*p*CO_2_ treatment ≈400 and 1200 μatm (for pHT and *p*CO_2_ respectively) with one control condition without any kind of CO_2_ manipulation, aiming to control the effect of experimental confinement on the composition of a natural bacterial community. In fact, the majority of the significant changes in taxonomic abundance and phylogenetic structure, particularly for the attached communities, were in response to containment. We were able to detect small shifts in phylogenetic structure and diversity that could have subtle effects on the maintenance of functionality in a complex community. New in situ tools need to be developed that could monitor such changes. For future studies, it is necessary to include the use of metatranscriptomic tools in order to understand if bacterial composition changes are related to changes in the functionality of bacterial groups.

## 5. Conclusions

The results of this study lead us to propose that changes in pH and *p*CO_2_ concentrations modulate the composition of bacterial communities being able to influence the biogeochemistry process of the ocean. The results of the present study provide insight into the vulnerability and resilience of microorganisms against changes resulting from low pH/high *p*CO_2_ conditions and provide some foundations for future studies aiming to evaluate the potential implications of such changes for biogeochemical cycles of highly relevant elements in the ETSP region off northern Chile.

## Supplementary Materials

The following are available online at https://www.mdpi.com/2076-2607/8/12/1924/s1. Figure S1: Nutrient measure in microcosm during 5 days of incubation. (A) NO_3_ (B) NO_2_ (C) PO_4_^3−^. Black circle represents oceanic water from station T5, Gray circle represents the control treatment, Blue circle represented high pH/low *p*CO2, Red circle represents treatment under low pH/high *p*CO2. Figure S2: Characterization of the environmental variables in microcosm. (A) pH (B) *p*CO_2_ (C) Cellular abundance. Black circle represents oceanic water from station T5, Gray circle represents the control treatment, Blue circle represented high pH/low *p*CO_2_, Red circle represents treatment under low pH/high *p*CO_2_. Figure S3: Cluster diagram of relative abundance of the most abundant taxa (above 1% in at least one sampling point in microcosm) assigned to bacterial phylogenetic groups obtained from station T5, Control, microcosms high pH/low *p*CO_2_ and microcosms under low pH/high *p*CO_2_ in two fraction size (A) Free-living and (B) Particle attached, *n* = 3. Table S1: BIO-ENV results show the combination of variables that best explain the particle attached bacterial community pattern. The left column contains the variables considered in the analysis and the right column contains the Spearman correlation coefficient (Rho). Table S2: BIO-ENV results show the combination of variables that best explain the free-living bacterial community pattern. The left column contains the variables considered in the analysis and the right column contains the Spearman correlation coefficient (Rho).
