# Supplementary material for: The Influence of pCO2-Driven Ocean Acidification on Open Ocean Bacterial Communities during A Short-Term Microcosm Experiment in the Eastern Tropical South Pacific (ETSP) off Northern Chile
Source: Microorganisms. 2020 Dec 4;8(12):1924. doi: 10.3390/microorganisms8121924 (PMC7761846; doi:10.3390/microorganisms8121924)
Supplement: Supplementary file 1 [file microorganisms-08-01924-s001.pdf]

Supplementary Figures

The influence of pCO$_2$ driven ocean acidification on open ocean bacterial communities during a short-term microcosm experiment in the Eastern Tropical South Pacific (ETSP) off northern Chile

Paulina Aguayo[1, 2*], Víctor L. Campos[3], Carlos Henríquez[2, 4], Francisca Olivares[2,4], Rodrigo De la Iglesia[5], Osvaldo Ulloa[2,4], Cristian A. Vargas[1, 2, 6]

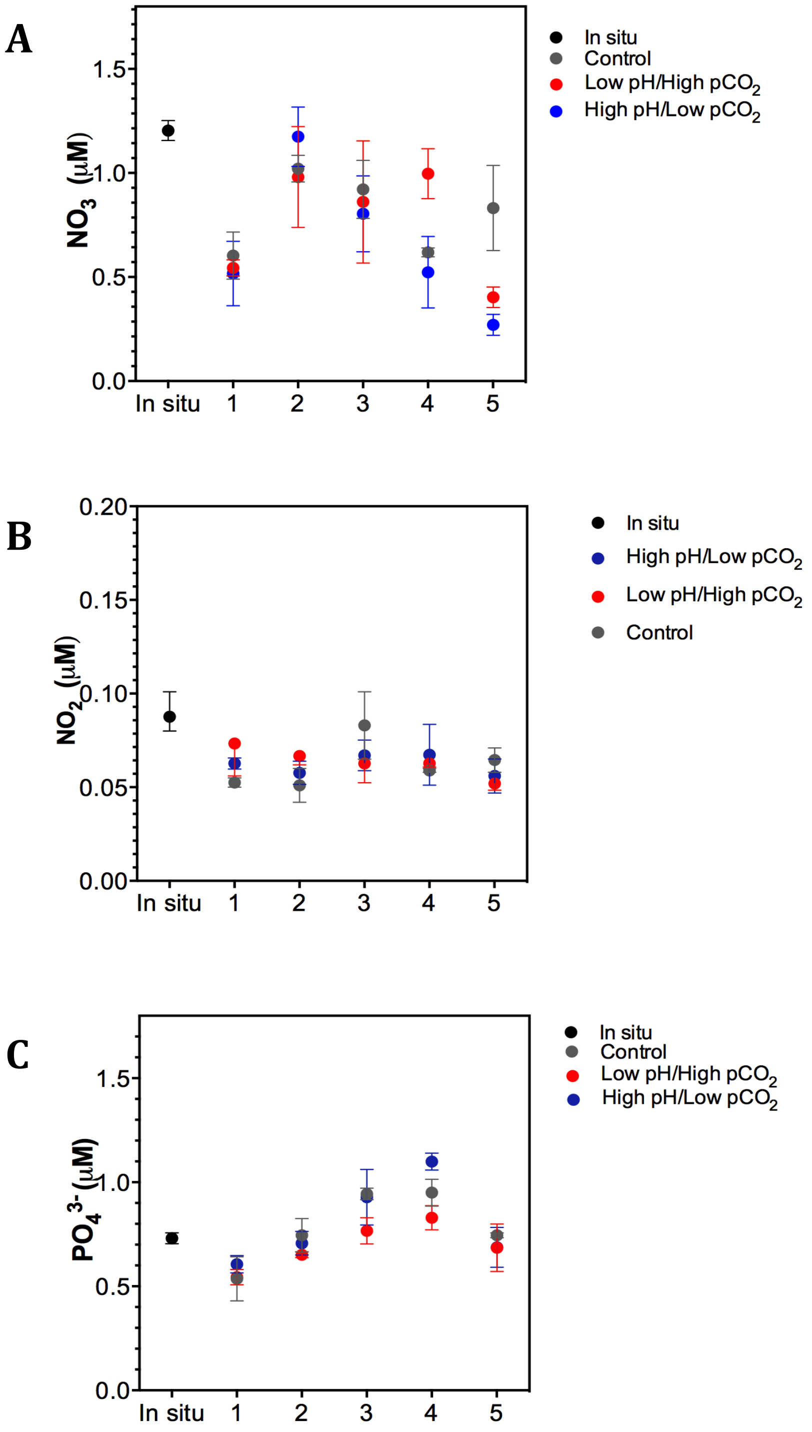

Figure S1. Nutrient measure in microcosm during 5 days of incubation. A) NO_3 B) NO_2 C) PO_4^{3-}. Black circle represents oceanic water from station T5, Gray circle represents the control treatment, Blue circle represented high pH / low pCO_2, Red circle represents treatment under low pH / high pCO_2

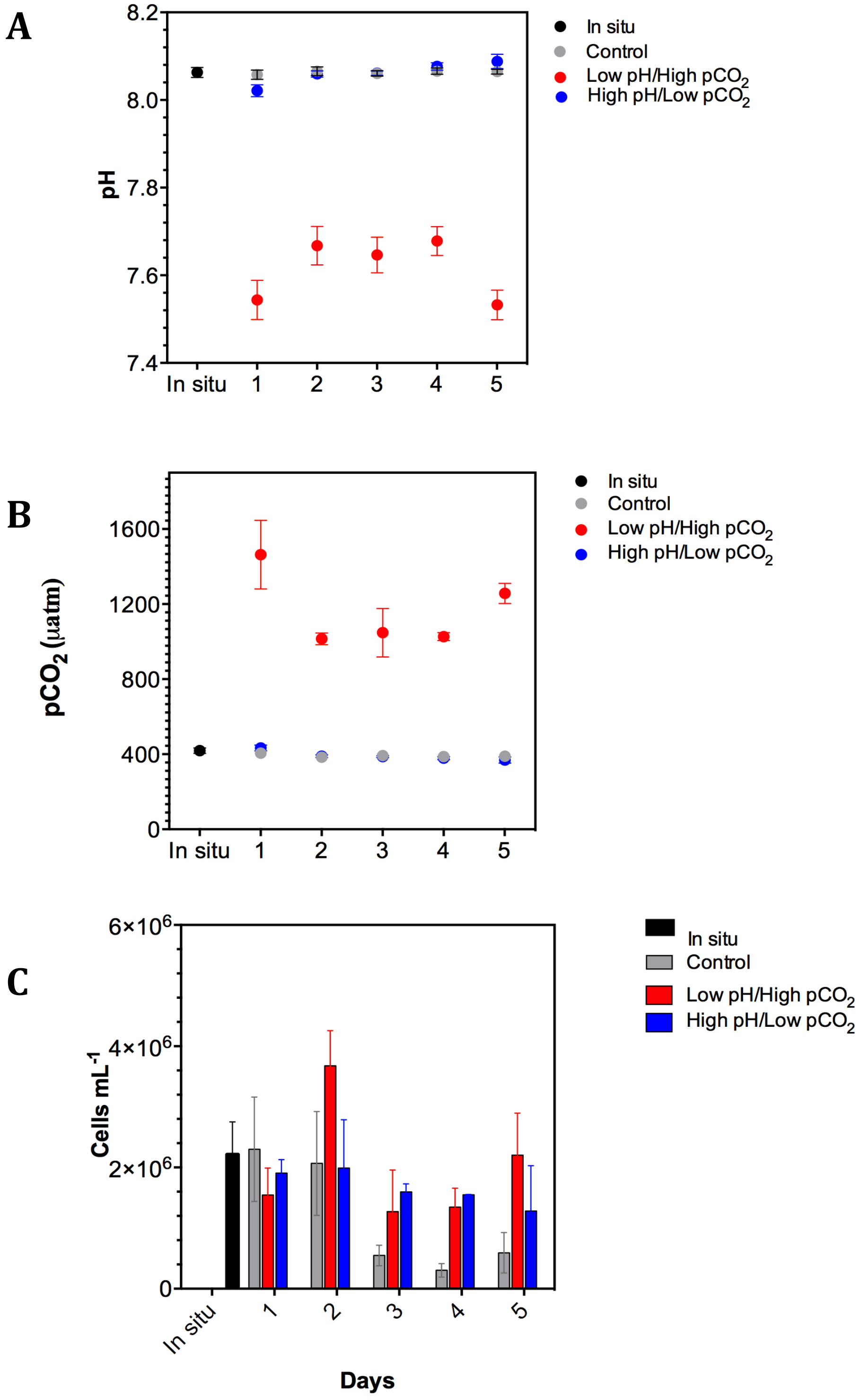

Figure S2. Characterization of the environmental variables in microcosm. A) pH B) pCO_2 C) Cellular abundance. Black circle represents oceanic water from station T5, Gray circle represents the control treatment, Blue circle represented high pH / low pCO_2, Red circle represents treatment under low pH / high pCO_2

A

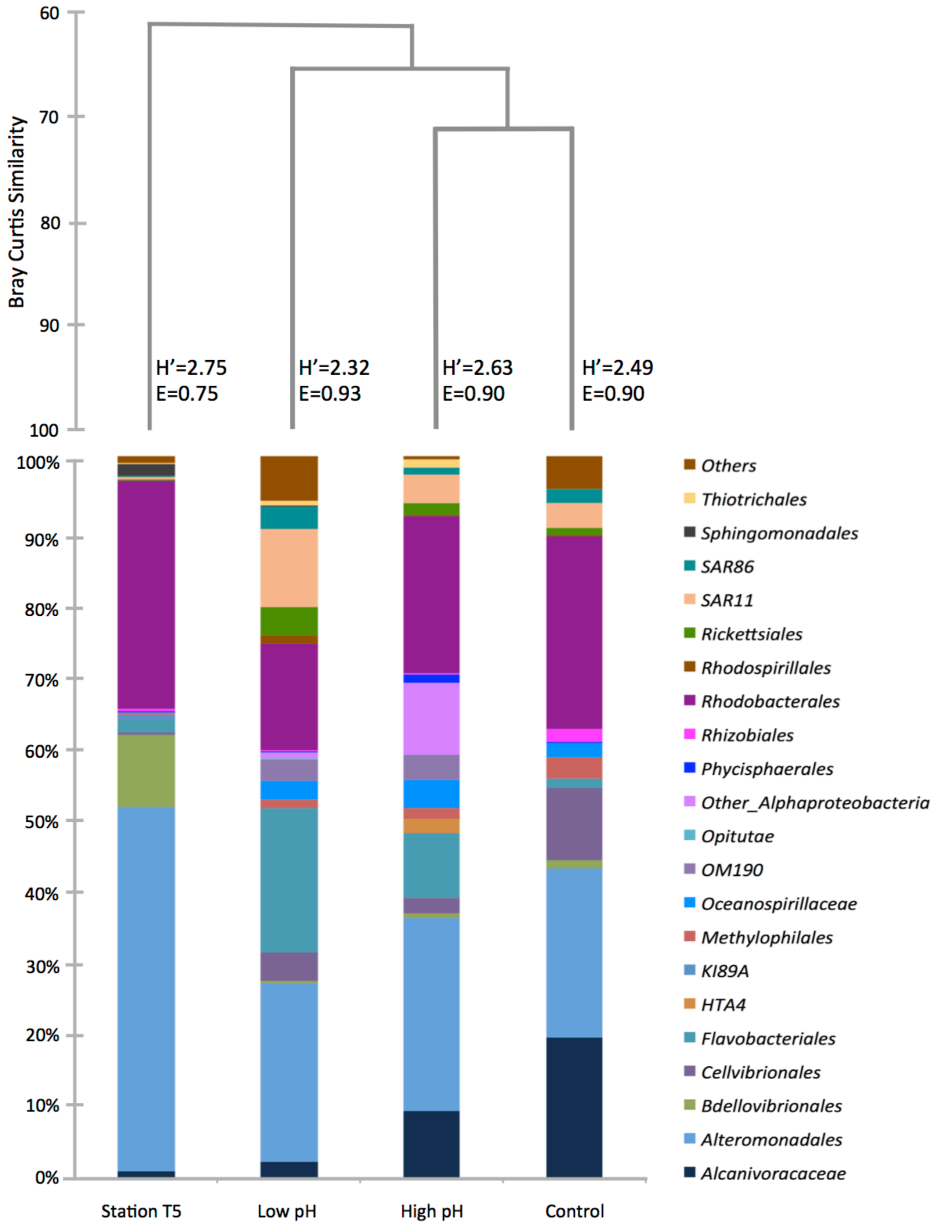

Bray Curtis Similarity
60
70
80
90
100
H'=2.75
E=0.75
H'=2.32
E=0.93
H'=2.63
E=0.90
H'=2.49
E=0.90
100%
90%
80%
70%
60%
50%
40%
30%
20%
10%
0%
Station T5
Low pH
High pH
Control
Others
Thiotrichales
Sphingomonadales
SAR86
SAR11
Rickettsiales
Rhodospirillales
Rhodobacterales
Rhizobiales
Phycisphaerales
Other_Alphaproteobacteria
Opitutae
OM190
Oceanospirillaceae
Methylophilales
KI89A
HTA4
Flavobacteriales
Cellvibrionales
Bdellovibrionales
Alteromonadales
Alcanivoracaceae

B

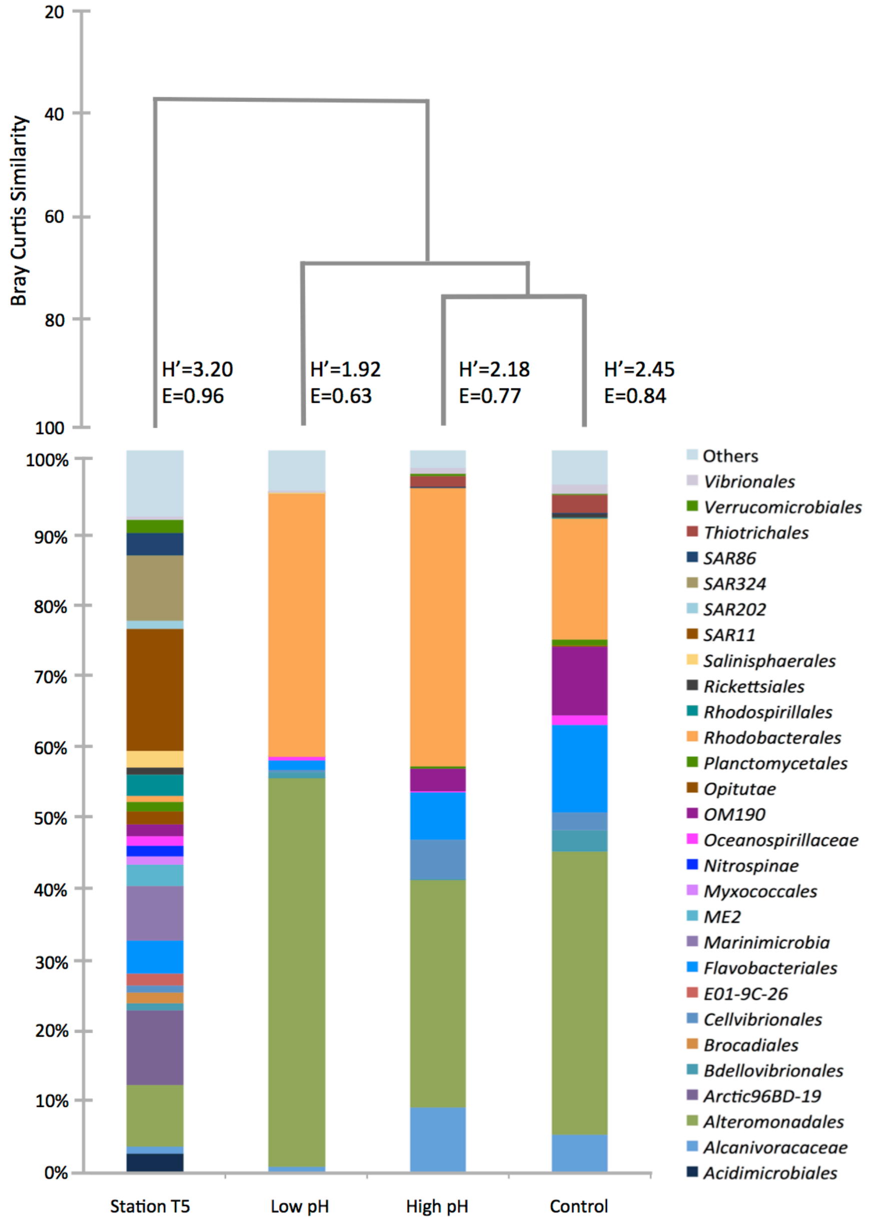

Figure S3. Cluster diagram of relative abundance of the most abundant taxa (above 1% in at least one sampling point in microcosm) assigned to bacterial phylogenetic groups obtained from station T5, Control, microcosms high pH/low pCO_2 and microcosms under low pH / high pCO_2 in two fraction size A) Free- living and B) particle attached), n=3.

Table S1: BIO-ENV results show the combination of variables that best explain the particle attached bacterial community pattern. In the left column are the variables considered in the analysis and in the right column the Spearman correlation coeficient (Rho).

Single Variable	**Rho**
Oxygen_CTD	0.647
Temp_CTD	0.614
Si	0.606
TA	0.573
Salinity_CTD	0.553
pH_AT_25	0.530
*p*CO2	0.516
P	0.444
Nitrite	0.152
Nitrate	0.081
DIC	0.051
Multiple Variable	**Rho**
Oxygen_CTD + P	0.683
Oxygen_CTD + P + Temp_CTD	0.683
Oxygen_CTD + P + Salinity_CTD	0.683
Oxygen_CTD + P + pH_AT_25	0.683
Oxygen_CTD + P + TA	0.683
Oxygen_CTD + P + DIC	0.683
Oxygen_CTD + P + Temp_CTD + Salinity_CTD	0.683
Oxygen_CTD + P + Temp_CTD + pH_AT_25	0.683
Oxygen_CTD + P + Temp_CTD + TA	0.683
Oxygen_CTD + P + Temp_CTD + DIC	0.683

Table S2: BIO-ENV results show the combination of variables that best explain the free-living bacterial community pattern. In the left column are the variables considered in the analysis and in the right column the Spearman correlation coeficient (Rho).

Single Variable	**Rho**
P	0.818
Nitrate	0.758
Si	0.740
Salinity_CTD	0.706
pCO2	0.687
Temp_CTD	0.661
pH_AT_25	0.638
TA	0.521
Oxygen_CTD	0.406
DIC	0.069
Nitrite	-0.032

Multiple Variable	**Rho**
Si + Nitrate	0.840
Si + Nitrate + Temp_CTD	0.840
Si + Nitrate + Salinity_CTD	0.840
Si + Nitrate + pH_AT_25	0.840
Si + Nitrate + TA	0.840
Si + Nitrate + P	0.840
Si + Nitrate + DIC	0.840
Si + Nitrate + Temp_CTD + Salinity_CTD	0.840
Si + Nitrate + Temp_CTD + pH_AT_25	0.840
Si + Nitrate + Temp_CTD + TA	0.840
